# Training secondary school students as anti-smoke ambassadors using the service-learning model: A cluster randomized controlled trial with hybrid type 1 effectiveness-implementation design study protocol

**DOI:** 10.1371/journal.pone.0313404

**Published:** 2024-11-14

**Authors:** Katherine Ka Wai Lam, Ka Yan Ho, Doris Yin Ping Leung, Arkers Kwan Ching Wong, Cynthia Sau Ting Wu, Charlotte Qi Liu, Ting Mao, Yim Wah Mak

**Affiliations:** School of Nursing, Hong Kong Polytechnic University, Hong Kong Special Administrative Region, China; Public Library of Science, UNITED KINGDOM OF GREAT BRITAIN AND NORTHERN IRELAND

## Abstract

**Background:**

Evidence shows that using the AWARD (Ask, Warn, Advise, Refer, Do-it-again) model with service-learning model in youngsters may be an appropriate strategy to refer young smokers for early smoking cessation services. Therefore this study aims to promote smoking cessation by training secondary school students as anti-smoke ambassadors (ASAs) with increased knowledge, skills and self-efficacy on smoking cessation and AWARD model using service-learning model.

**Methods:**

A cluster randomized controlled trial will be conducted in 14 secondary schools in Hong Kong. Also, a hybrid type 1 effectiveness-implementation design with the Reach Effectiveness Adoption Implementation Maintenance (RE-AIM) framework will be adopted. For the intervention group, participants (n = 184) will attend a 3-hour training workshop, followed by hands-on sessions supervised by trained nursing students, then a 6-month smoker referral competition and an award presentation ceremony. The control group (n = 184) will only attend the 3-hour training workshop. The research assistant will contact participants at the start and the end of training program, and at 3, 6, and 12 months after the end of the training program by telephone to complete a set of questionnaires. Participating secondary schools, participating nursing students, ASAs, and responsible organizational staff will be randomly invited for a one-to-one semi-structured interview. The primary outcome will be the number of smokers who referred by secondary school students. Descriptive statistics, mixed between-within-subjects ANOVA, logistic regressions, and content analysis will be used.

**Discussion:**

This study will bridge the gap in existing literature by determining the effectiveness and exploring facilitators and barriers in implementing our intervention with the use of the AWARD model and service-learning model in training young people to refer smokers to anti-smoke organizations in the real-world. This can enhance our community capacity and enable youngsters to take a proactive role to support smoking cessation.

**Trial registration:**

ClinicalTrials.gov, NCT05897346. Registered on 11 May 2023.

## Introduction

Smoking remains the leading cause of preventable premature death and illness worldwide [1], accounting for more than 8 million deaths annually [1, 2]. Evidence shows that smoking cessation interventions in young people are crucial for the tobacco endgame [3, 4], especially as smoking behavior from youth continues into adulthood [5]. Early smoking cessation interventions are pivotal.

Many young smokers who joined smoking cessation services were recruited through outreach activities and referrals from non-governmental organizations (NGOs) [6, 7]. Importantly, at the time joining the smoking cessation services, around half have already smoked for 4 years or more [6]. Given that early interventions are important for tobacco endgame [3, 4], more methods for recruitment has to be considered. Evidence shows that youngsters encounter peer smokers frequently and easily in their social network [5–8]. Building the community’s capacity by training youngsters to refer smokers to professional smoking cessation services therefore tend to offer a good strategy to support smoking cessation. Additionally, the trained youngsters will be educated about not smoking.

Studies also show that peer pressure is considered the most common reason for smoking in young people [5, 8]. Smoking cessation interventions that target peers are likely to have substantial effects. The second most common reason reported is curiosity [8]. This suggests that if young people clearly understand the consequences of smoking, they may be less likely to smoke because of curiosity. Evidence also indicates that youngsters may not have adequate knowledge about smoking and its consequences, which results in myths about smoking [9]. Once smoking behavior is initiated, individuals are easily addicted, which results in difficulty in quitting, thereby carrying smoking behavior into adulthood and later life [5]. Therefore, education to resolve these myths is particularly important.

Apart from resolving the myths in youngsters, a literature review revealed that brief interventions and referrals are important strategies to assist smokers to receive professional help to quit smoking successfully, as supported by the World Health Organization (WHO) and previous publications [10–12]. Particularly, the AWARD (Ask, Warn, Advise, Refer, Do-it-again) model emphasizes referrals and offers a brief and effective intervention for smoking cessation [11]. For instance, the Council on Smoking and Health and the Education Bureau in Hong Kong conducted the Smoke-free Elite Teens Program (formerly known as the Smoke-free Teens Program) [13, 14]. This program aims to nurture young people aged 14–18 years to promote a smoke-free culture in Hong Kong by firstly teaching participants the AWARD model and updated information on tobacco control, then requiring them to organize smoke-free programs in the community [13, 14]. The program has gained support from over 160 secondary schools, youth centers, and uniform groups. Nevertheless, the referrals rate was low with 1% of participants had referred smokers to smoking cessation services after the program [13]. One key possible reason is that the program did not provide hands-on experience to participants to proactively approach smokers and use the AWARD model. Some reported that they felt embarrassed and did not know how to effectively advise smokers to quit or refer smokers to professional services. Evidence also shows that hands-on experience is needed to effectively deliver learned skills [15].

In order to provide hands-on experience to youngsters, using service-learning model may be a suitable strategy [7, 16]. Service-learning models are used in some existing smoking cessation services such as Youth Quitline (YQL) [7]. YQL [7, 17] is a telephone hotline funded by the Department of Health in Hong Kong to train undergraduate nursing students to provides free peer smoking cessation counseling to young smokers aged 25 years or below. The quit rate in YQL is high [6], which represents a major success when compared with earlier results and other smoking cessation services in Hong Kong [6, 18]. YQL may be an appropriate venue for youngsters to gain hands-on experience of the AWARD model.

Hence our proposed study based on the service-learning model will provide opportunity for young people to learn and practice the AWARD model with hands-on experience under the supervision of nursing students who are considered peer counselors in YQL. However, no previous study has been conducted to explore the effectiveness, facilitators and barriers of our intervention in training young people to refer smokers to anti-smoke organizations in the real-world. Implementation study will be required to investigate facilitators and barriers of the interventions apart from the effectiveness, aiming to promote the systematic uptake of interventions in the real-world and hence improve the quality and effectiveness of health services [19].

### Theoretical models

#### AWARD model

The AWARD model involves an effective five-step process for smoking cessation when an individual encounters smokers: 1) Ask about smoking history, 2) Warn about the high risk of smoking, 3) Advise to quit; 4) Refer smokers to smoking cessation services; and 5) Do it again [11]. It emphasizes referral (i.e., referring smokers to existing smoking cessation services), which has been found to assist smokers to quit successfully [11]. This model is particularly suitable for young people as it is easily learned and used with minimal training [11]. Additionally, it takes only a minute or slightly longer to deliver smoking cessation advice based on the AWARD model [11]. Thus a large number of smokers can be reached at low cost [11].

Brief interventions using the AWARD model have been found to effectively assist smokers to quit and build community capacity for smoking cessation. Our previous study trained 50 girl guides to promote smoking cessation among community-living female smokers using the AWARD model which only lasted for a minute or slightly longer [11]. The findings showed that the intervention was feasible and effective in promoting smoking cessation among community-living female smokers [11].

#### Service-learning model

In this study, the service-learning model provides young people with opportunities to gain hands-on experience in using the AWARD model. The service-learning model has been used by universities over the past decade in teaching [20]. Service-learning is a type of experiential education that includes two fundamental constructs: service and learning [20]. The model requires senior or experienced workers who can monitor and supervise students in conducting activities when serving the community. It has been documented to benefit students by facilitating their intellectual and academic development and building their sense of civic responsibility through reflections and applying knowledge learned in classroom to real-world practice [20]. The model has also been published in our previous publication [7] and is currently being used to operate YQL. Under this model, nursing students are trained as peer counselors and receive supervision by nursing teachers while delivering smoking cessation counseling in their clinical placement in YQL.

### Aim and objectives

This study aims to promote smoking cessation by training young people as anti-smoke ambassadors (ASAs) with increased knowledge on smoking cessation and skills using the AWARD model to build community capacity in smoking cessation.

The objectives are to: 1) examine the effectiveness of our program in assisting secondary school students to refer smokers to YQL (primary objective), 2) increase ASAs’ knowledge about smoking cessation and skills using the AWARD model, 3) enhance their practice and attitudes toward smoking cessation and AWARD model, 4) increase their self-efficacy in using AWARD model skills, 5) provide hands-on experience in using the AWARD model through the service-learning model, 6) train ASAs to deliver brief interventions to young smokers using the AWARD model, and 7) identify facilitators and barriers in implementing in the real-world.

## Materials and methods

**Fig 1:**
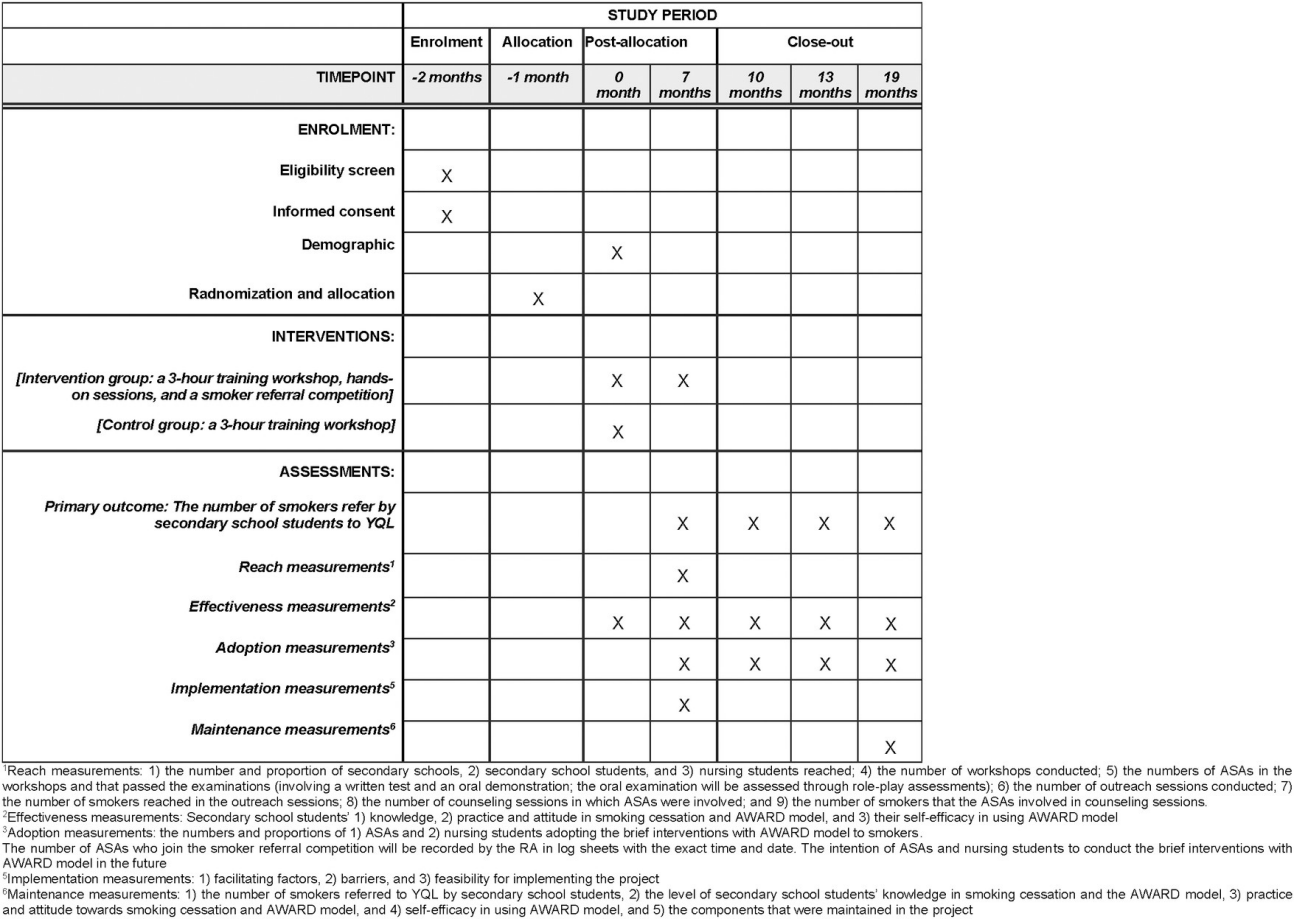
Enrolment, intervention, and evaluation schedule for SPIRIT. The schedule of enrolment, interventions, and assessments for SPIRIT.

The recruitment of this study was started in 1 August 2023 and the tentative end date of the recruitment is 31 December 2024. The study protocol is consistent with the Standard Protocol Items: Recommendations for Intervention Trials (SPIRIT) (see S1 File). The schedule of enrolment, interventions, and assessments for SPIRIT is presented in Fig 1.

### Study design

**Fig 2:**
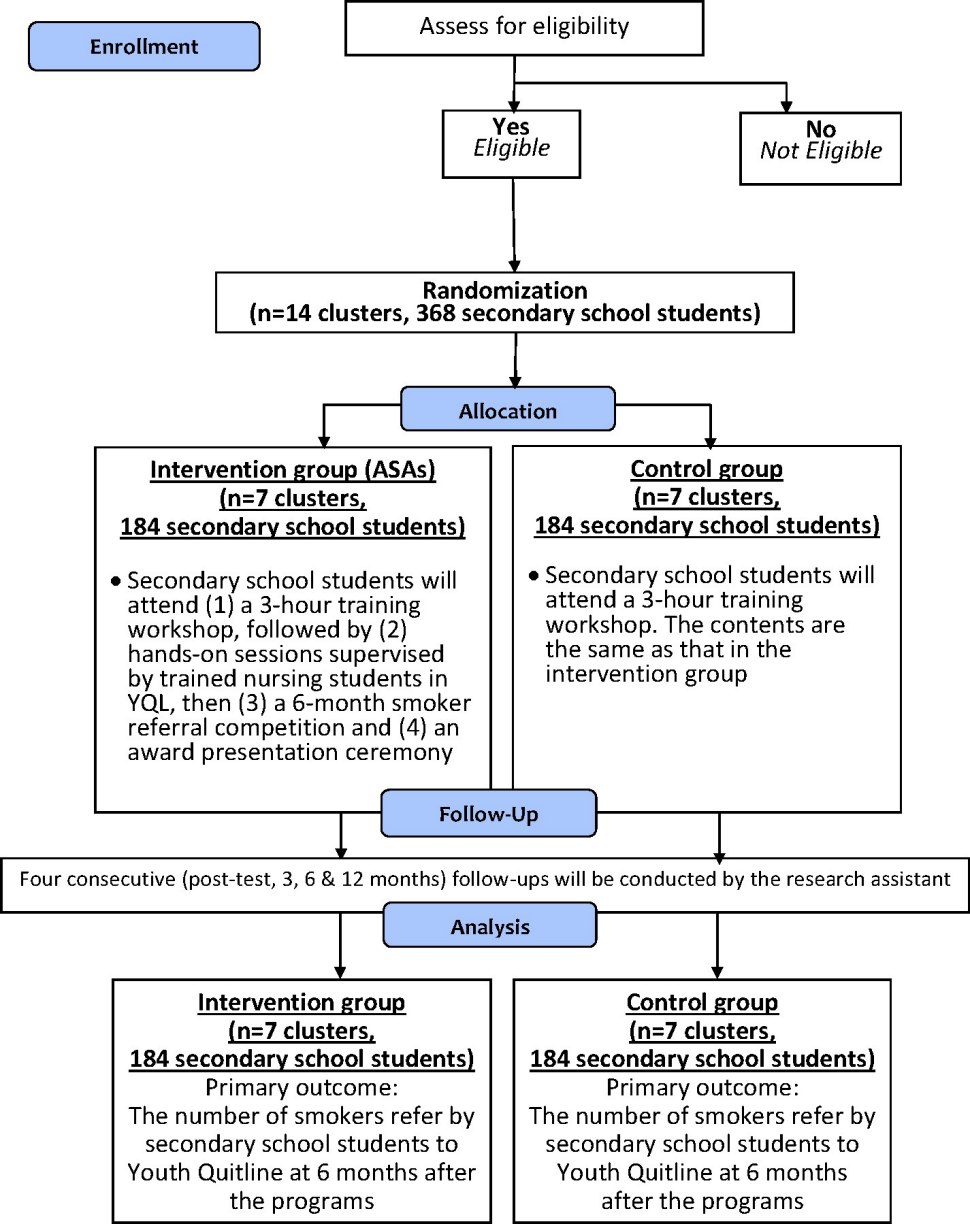
CONSORT flow diagram. A recommended diagram to illustrate the flow of randomized controlled trials.

A hybrid type 1 effectiveness-implementation design will be adopted as it can primarily focus on testing the effectiveness of our intervention on the relevant outcomes while observing and gathering information on implementation [21]. This can allow us to identify barriers, facilitators, and what is required for supporting the implementation in the real world and therefore inform the appropriate implementation strategies [21]. With the hybrid type 1 design, our main objective is to examine the effectiveness of the intervention. Information relating to implementation will also be studied as secondary outcomes. To do so, a two-arm cluster randomized controlled trial (RCT) following the CONSORT statement in Fig 2 will be conducted to examine the effectiveness of the program in referring smokers to YQL.

### Study setting

This is a 3-phased study conducted in 14 secondary schools in Hong Kong.

### Clusters and randomization

14 clusters (14 schools) will be randomized to the intervention or control group using a 1:1 ratio by a research assistant (RA) who is not from our team using an independent computerized randomization sequence process that can only be accessed by that RA to ensure allocation concealment. This RA will login to an online platform to perform randomization. Randomization sequences will be generated by the computer in randomly permuted blocks sizes of 2, 4, and 6. The RA responsible for recruitment will record the exact date and time of recruitment. Computerized time stamps and electronic logs of sent allocation will be used to ensure allocation concealment. The RA undertaking recruitment will be responsible for data collection and be blinded to the group assignment.

### Eligibility criteria

Secondary school students (Forms 2–4) who can understand Cantonese are the target group as most daily smokers started smoking weekly at age 10–19 years [8]. Those with identified cognitive or behavioral problems that affect their ability to respond to questionnaires will be excluded.

### Sample size calculation

G*Power [22] was used based on a small-to-medium effect size on the primary outcome, i.e. number of smoker referred by secondary school students at the 6-month follow-up (d = 0.35), based on our team’s experience in smoking cessation projects [11–13]. Hence, to detect a significant difference in the number of smoker referred between the two groups with a significance level of 5% and power of 80%, a total of 260 secondary school students would be needed. Based on a conservative estimation, around 360 eligible secondary school students can be approached in each school. The response rate for previous workshops of a similar duration was around 8% conservatively [11, 23]. Hence, we expect to recruit 28 (360 × 0.08) students in each school. For cluster RCT, it is estimated that the intracluster correlation coefficients = 0.01, and the design factor = (1+(28–1)*0.01) = 1.27, then the sample size needed will be 331 (260*1.27). With an estimated 10% attrition rate at 12 months, the total sample size needed will be 368 students (331/0.9) with 184 students in each group. Thus, a total of 14 schools with 7 schools (184/28) in each group are needed.

### Intervention group (ASAs)

#### Phase 1: ASA recruitment

This involves establishing a network for smoking cessation in the community. It includes recruiting ASAs through secondary schools. Promotional leaflets will be designed and distributed to the two NGOs and other secondary schools.

#### Phase 2: Training program

The training program will involve **1) training workshops**, **2) hands-on sessions,** and **3) a smoker referral competition**.

**Fig 3:**
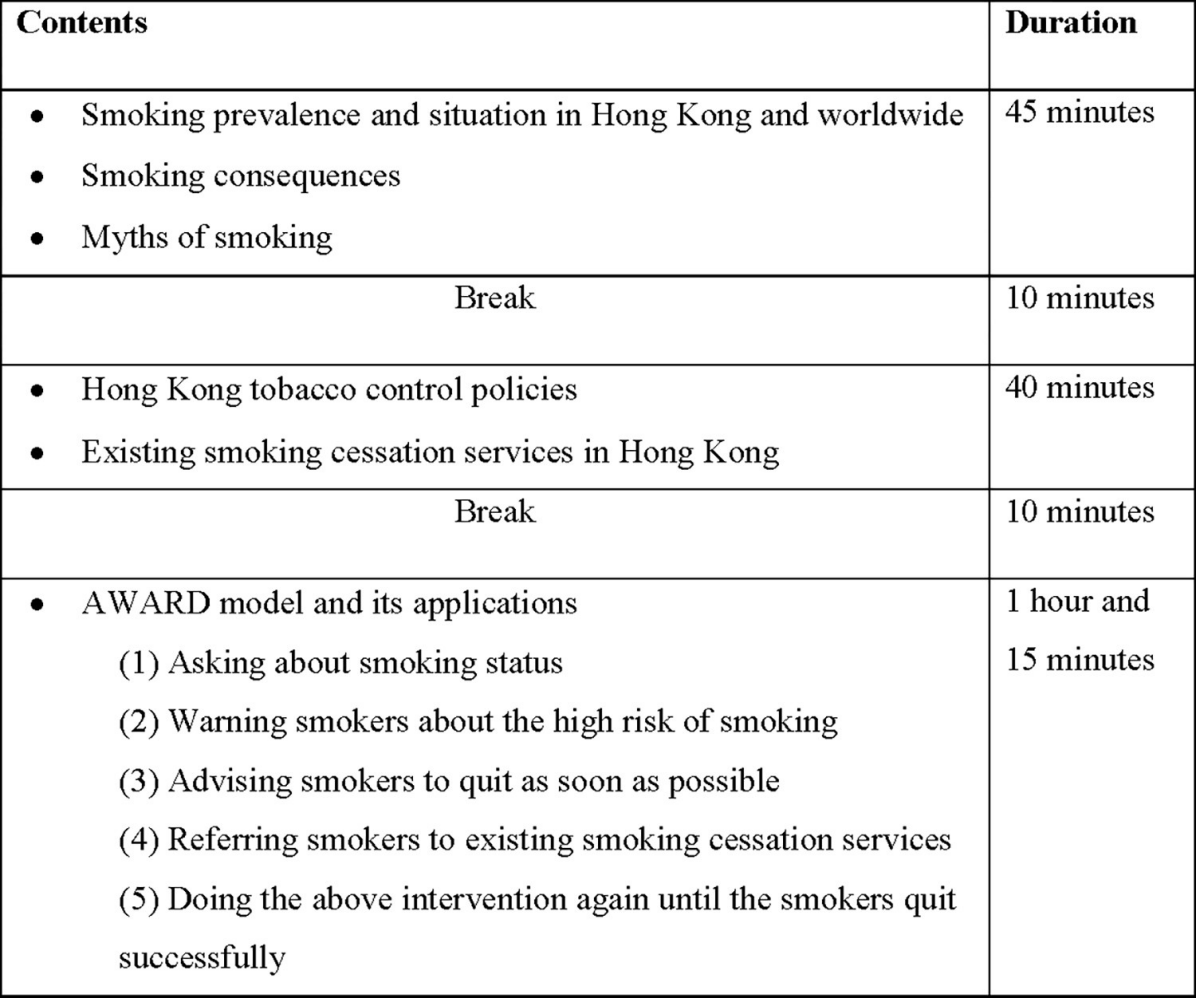
The rundown of the workshop. A figure showing the details of the workshops’ rundown.

**Fig 4:**
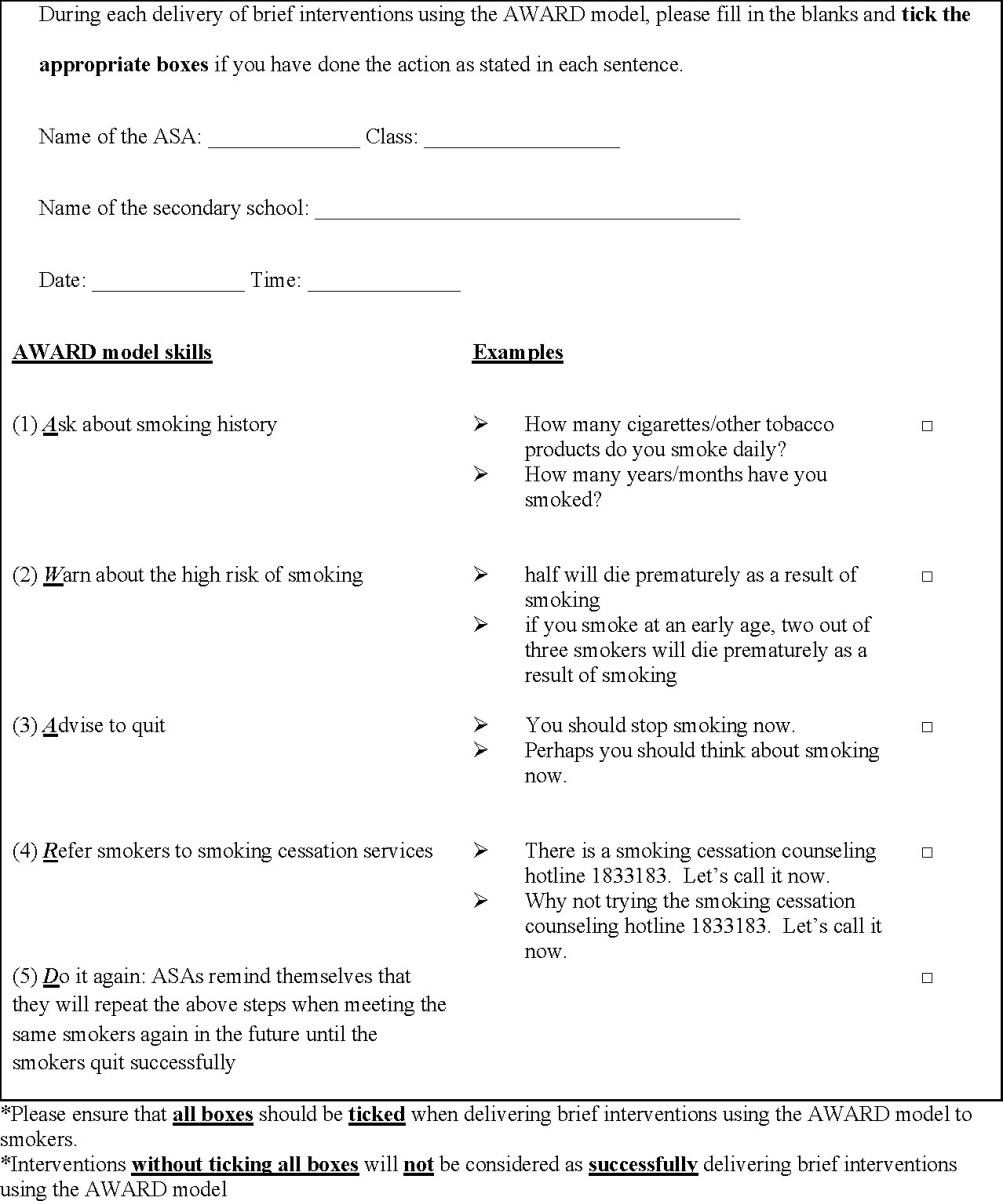
Checklist of the brief interventions using the AWARD model. The template of a checklist for ASAs to use in each intervention delivery.

*1*. *Training workshops*. A half-day workshop (Fig 3) will be conducted by an RA trained by our team to train ASAs. Each workshop will last for 3 hours in secondary schools, at a local University, or online (depending on the preference of schools). The workshops will be based on those implemented in our previous smoking cessation projects [6, 7, 11, 13]. Specifically, when teaching the AWARD model, we will focus on educating ASAs about the skills needed to assess smokers’ smoking status, readiness to quit, and nicotine dependence. Importantly, ASAs will be taught how to deliver a brief intervention using the AWARD model (lasting about a minute or slightly longer) (Fig 4) [11]. Information of YQL will be highlighted so that ASAs can invite smokers to call YQL in ‘***R***efer’ of the AWARD model [11]. Also in ‘***R***efer’ of the AWARD model, the ASAs will be trained with some safety tips by the RA, including 1) stay with the respective nursing students at all times during the outreaching sessions, 2) stay on main streets only and never go to alleys, 3) never reveal your identity cards/student cards/telephone numbers or other documents/numbers that reveal your identities, 4) report to nursing students if you feel unwell/ any discomfort, 5) introducing signals of discord, and 6) educating appropriate solutions dealing with signals of discord [24].

Each ASA will be required to pass an examination before proceeding to the hands-on sessions. This examination has been adopted in our previous projects [6, 7, 11]. It will first involve a written test asking ASAs about their knowledge of smoking consequences and AWARD model, then an oral demonstration in response to a case scenario of a smoker (role-played by nursing students). The examinations will be assessed by our RA and team members, with 50% as the passing mark. Those who fail will be required to rejoin the training workshops and retake the examination until a passing mark is obtained.

*2*. *Hands-on sessions*. ASAs will then be required to follow their assigned nursing students to implement the brief interventions to smokers in Hong Kong. Specifically, nursing students will bring their ASAs to the outreach activities (2 hours each) to supervise ASAs delivering brief interventions using the AWARD model when encountering smokers aged 25 or below smoking. Each ASA will follow their respective nursing student to four outreach activities and two counseling sessions (2 hours each). During the counseling sessions, ASAs will listen to how the nursing students deliver smoking cessation counseling and join the conversations by applying the AWARD model, offering ASAs opportunity to experience smoking cessation counseling. All ASAs will receive a certificate after completion of this phase.

*3*. *Smoker referral competition*. All ASAs will then be given a 6-month period to use the AWARD model to deliver brief interventions to refer smokers who are aged 25 years or below to YQL independently, without the help of nursing students. To ensure ASAs’ safety, they are asked to approach smokers via their social network including peer smokers, smokers in families and schools. Smokers are defined as those who smoked tobacco products (including electronic cigarettes) in the previous 30 days, as defined in YQL and other projects related to smoking [6, 11].

Near the end of the brief intervention with the AWARD model, ASAs will provide the contact of YQL and invite the smokers to call YQL. Those who successfully contacted YQL staff will be regarded as a successful referral case.

#### Intervention fidelity

To ensure consistency of the brief interventions, all ASAs will be asked to adhere to the intervention protocol and complete the checklist developed by our team (Fig 4) after each delivery of the brief intervention. Additionally, each ASA will be supervised by a trained nursing student. ASAs will be encouraged to share any difficulties in delivering the brief interventions to the responsible nursing student for discussion and feedback. During the competition, ASAs will also be recommended to consult our RA if they encounter any problems. This study will also include audit trial which is independent from our team members and funder.

#### Safety issues

A YQL staff will also be present in each outreaching activity to monitor the students and provide assistance when necessary. Both nursing students and ASAs will be given a contact of the PI for emergency contact.

#### Phase 3: Award presentation ceremony

10 award certificates will be given to the top 10 ASAs who deliver brief interventions using the AWARD model to the largest number of smokers (excluding smokers found with the nursing students). The champion will receive a HKD4000 book coupon. The first- and second- runner up will respectively receive HKD 3000 and HKD2000 book coupons. The remaining 7 ASAs will each receive a HKD1000 book coupon. The incentives are also expected to improve the adherence of this study.

### Control group

Secondary school students will be only required to attend a 3-hour training workshops with AWARD model in their schools or the University by our trained RA. The contents of the workshops are the same as that in the intervention group. However control group will not join the examinations, hands-on sessions, the smoker referral competition nor be given checklists for conducting the brief interventions with AWARD model.

### Data collection

After randomization, responsible teachers in schools will first be briefed by our RA about the purpose and protocol of the project. The schools will then distribute our promotional leaflets, information sheet and consent forms to students or parents depending on schools’ preferences. Consent forms with students’/parents’ telephone numbers will be returned to teachers if students are interested in joining the project. Eligible students will be informed by our RA via telephone that their participation will be voluntary and confidentiality will be maintained. They will also be notified about the details and schedule of the program activities.

The RA will contact the students in the intervention and control group at the start of training program (T1), at the end of training program (T2), and at 3 (T3), 6 (T4), and 12 months (T5) after the end of the training program by telephone to complete a set of questionnaires.

At T2, each ASA will submit a 300-word reflective journal. Responsible teachers in participating secondary schools, participating nursing students, ASAs, and responsible YQL staff will be randomly invited to complete a one-to-one semi-structured interview focused on implementation. The interviews will be conducted by an RA at T2 until data saturation is reached.

At T5, ASAs and nursing students will be interviewed about maintenance. Another RA who is not involved in this project will be asked to generate random numbers using a computer for random selection of participants for the interviews. All interviews will last for around 15–30 minutes and be audio-recorded in a room in our university or online (e.g., via Zoom) depending on the preference of interviewees. Based on the interview guide developed by our team, they will be asked about their overall experience, facilitating factors, barriers, and feasibility of implementing brief interventions using the AWARD model to smokers at T2. At T5, they will be asked about the maintained effects and components of the project. It is estimated that around 40 and 20 interviewees will be needed to reach data saturation at T2 and T5, respectively. Each interviewee will receive HKD$200 as a travelling allowance.

### Outcomes

The primary outcome will be the number of smokers who referred by secondary school students to YQL at T4 (at the end of the 6-month competition). Secondary outcomes include the reach, adoption, implementation, maintenance, and other effectiveness measurements.

### Measurements

1. Demographic data including age, sex, household size, household income, living district, and smoking status of the secondary school students and participating nursing students will be collected at T1.
  The Reach Effectiveness Adoption Implementation Maintenance (RE-AIM) framework will be adopted [25].
2. Reach will be recorded as: 1) the number and proportion of secondary schools, 2) secondary school students, and 3) nursing students reached; 4) the number of workshops conducted; 5) the numbers of ASAs in the workshops and that passed the examinations (involving a written test and an oral demonstration; the oral examination will be assessed through role-play assessments); 6) the number of outreach sessions conducted; 7) the number of smokers reached in the outreach sessions; 8) the number of counseling sessions in which ASAs were involved; and 9) the number of smokers that the ASAs involved in counseling sessions. Measurements from 7) to 9) will be recorded in log sheets with the exact time and date by ASAs. Other numbers will all be recorded in log sheets with the exact time and date by the RA.
3. Effectiveness will be recorded in terms of: 1) the number of smokers refer by secondary school students to YQL (primary effectiveness measure), 2) the level of secondary school students’ knowledge in smoking cessation and AWARD model (will be assessed by asking ASAs to respond “True/False/Don’t know” to 19 related statements at T1–T5), 3) practice (such as asking the secondary school students the number of times delivering the brief intervention and the number of smokers who receive their brief interventions) and attitudes toward smoking cessation and AWARD model (will be recorded by asking ASAs to respond to 31 related items at T1–T5), 4) self-efficacy in using AWARD model (will be assessed by asking ASAs to answer 3 items (scale 1–10) at T1–T5). The scales have all been used in various smoking cessation projects [7, 11, 13, 14].
4. Adoption: the numbers and proportions of 1) ASAs and 2) nursing students adopting the brief interventions with AWARD model to smokers. The number of ASAs who join the smoker referral competition will be recorded by the RA in log sheets with the exact time and date. The intention of ASAs and nursing students to conduct the brief interventions with AWARD model in the future at T2-T5 will also be assessed.
5. Implementation: 1) facilitating factors, 2) barriers, and 3) feasibility for implementing the project. These factors will be explored using semi-structured interviews with the responsible teachers in the participating secondary schools, ASAs, participating nursing students, and responsible staff in YQL at T2.
6. Maintenance, or the effectiveness of the project in long term will be measured at T5: 1) the number of smokers referred to YQL by secondary school students, 2) the level of secondary school students’ knowledge in smoking cessation and the AWARD model, 3) practice and attitude towards smoking cessation and AWARD model, and 4) self-efficacy in using AWARD model. At the setting level, the components that were maintained in the project at T5 will be assessed using semi-structured interviews with ASAs and participating nursing students.

### Data analysis

Quantitative data will be analyzed using SPSS. Descriptive statistics will be reported (objectives 5 and 6). The characteristics of ASAs and nursing students who intend to use the AWARD model in the future will be compared with those who do not (adoption) using independent sample t-tests and chi-square tests (objective 6). Mixed between-within-subjects analysis of variance and odds ratios with logistic regression will be used to examine the differences in outcomes between the ASAs and the control group (objectives 1–4) at each follow-up. Intention-to-treat analyses and multiple imputation will also be used.

QSR NVivo will be used to organize the qualitative data from the interviews (objective 7) and reflective journals. ASA’s reflective journals will be particularly analyzed for the effects of our program in helping ASAs to refer smokers to YQL, ASAs’ level of knowledge, practice and attitude in smoking cessation and the AWARD model, and self-efficacy in using the AWARD model (objective 1–4). Content analysis [26] will be performed by two authors (KWKL and KYH) separately by applying bracketing. Descriptions will be returned to participants for checking and feedback. Any discrepancies will be discussed and resolved in regular meetings among team members.

### Data management

All data will be stored in the University. KWKL who is principal investigator of this study will take the primary role to maintain the integrity and accuracy of data entry process. To ensure the confidentiality of participants, all data and files will be encrypted with passwords and only be able to be accessed by our team members (KWKL, KYH, DYPL, ACKW, CSTW, CQL, TM, and YWM). All data will be anonymous while an identifiable code will be given to each participant. Hardcopies of data will all be stored in a locked cabinet in the University and been accessible by our team members. Our team members will assist in monitoring the whole data entry progress and discuss to resolve any ambiguities.

### Ethics consideration

This study has been approved by the Institutional Review Board of Hong Kong Polytechnic University (ref. HSEARS20221124003) (see S2 File). This study has been registered at ClinicalTrials.gov (NCT05897346). Information sheet will be provided before obtaining written consent. Participants and parents will be told that their participants are totally voluntary and there will be no penalty if they withdraw from the study at any time will be possible without penalty. All the participant data will be kept confidential.

**Fig 5:**
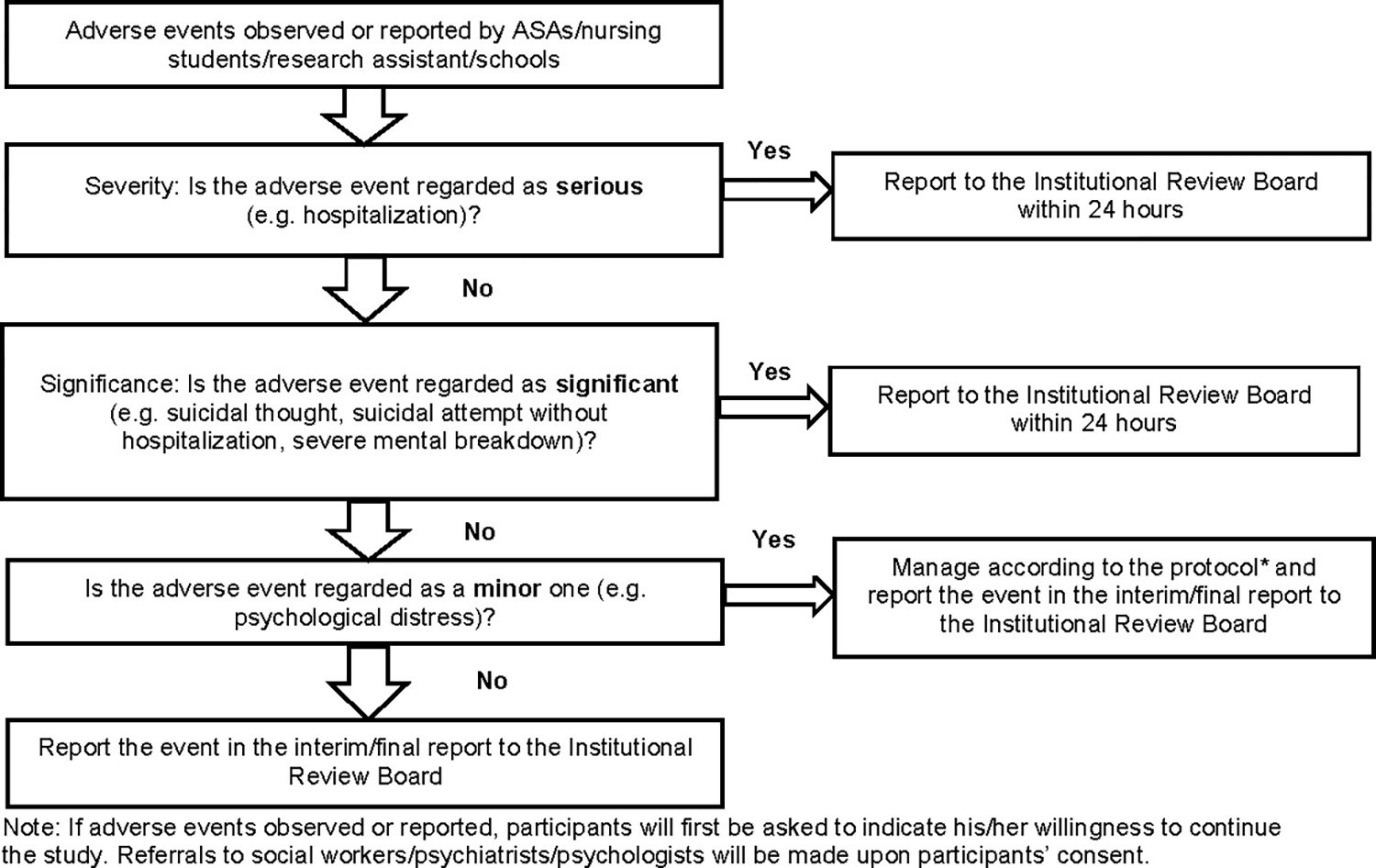
Protocol for adverse events management. A protocol for managing adverse events.

Although the chance of occurring adverse events during this study is expected to be minimal, a protocol for adverse events management is developed by our team to make referrals to social workers or psychiatrists or psychologists upon participants’ consent (Fig 5).

## Discussion

This study will bridge the gap in existing literature by determining the effectiveness and exploring the facilitators and barriers in implementing our intervention with the use of the AWARD model and service-learning model in training young people to refer smokers to anti-smoke organizations in the real-world. Although AWARD model has been found to be an effective and brief strategy to assist smokers to quit [11], hands-on experience from service-learning model may enable youngsters to effectively deliver brief interventions using the AWARD model to assist referring young smokers successfully to smoking cessation services. Therefore, building the community’s capacity by training youngsters under the service-learning model to refer smokers using the AWARD model may offer a good strategy to support smoking cessation. Using the implementation study design enables us to investigate facilitators and barriers of the interventions apart from the effectiveness, aiming to promote the systematic uptake of interventions in the real-world [19].

Importantly, this study can also enable youngsters to take a proactive role to support and perform ***O*** in MPOWER (i.e. ***O***ffer help to quit tobacco use) [27]. MPOWER are measures developed by WHO to assist countries to implement and manage tobacco control [27]. By training nursing students and youngsters who are our next generation, our community capacity for smoking cessation can be largely increased. Both nursing students and youngsters are highly encouraged to actively deliver anti-smoke messages to our whole community and importantly, to teach their families, friends or even future colleagues about what they have learnt in this study, further enlarging the community’s capacity in smoking cessation. After this study, not only secondary schools but also the primary school students and even the tertiary school students will be invited to join the intervention in the real-world setting. Our study findings will also be disseminated through media, publications, and conferences so as to raise public awareness in smoking cessation and tobacco endgame, inform the local and non-local health care systems and policy about the effectiveness of our intervention which can be highly considered to be incorporated into the current health care systems and policy. Hence, in the long run, it is expected that a large number of youngsters with smoking cessation knowledge who will not smoke can be trained with AWARD model skills to continuously refer smokers to existing smoking cessation services to quit smoking. With more smokers quitting and less youngsters starting smoking, the smoking prevalence and thereby the smoking-related health cost can be greatly reduced.

### Limitations of the study

One limitation is that blinding is not possible in this study. Second, this study can only provide follow-ups up to 12 months. Longer follow-ups may help explore the long-term impact of our intervention on participants in the real-world setting. Third, attritions will be expected. However, our sample size estimation has increased the sample size to a potential loss to follow-up up to 10%. Finally, the finding of this study is only applicable in smoking cessation but not in other addictive behaviors. However, it is expected that future studies may use our finding as the foundation to explore the possibility in implementing in other addictive behaviors.

## Conclusion

This study is expected to address the literature gap by conducting an implementation science study to explore the effectiveness, facilitators, and barriers in implementing an intervention with the use of the AWARD model and service-learning model in training young people to refer smokers to anti-smoke organizations in the real-world. The finding is anticipated to provide evidence of such an intervention to enhance referrals of smokers in the real-world. Through training youngsters with hands-on experience of using the AWARD model under the service-learning model, youngsters are also expected to learn to take a proactive role to support smoking cessation, hence strengthening our community capacity for smoking cessation.

## Supporting information

S1 File SPIRIT checklist. Recommended items to address in a clinical trial protocol and related documents.
(DOCX)

S2 File Ethics approval. The letter of notification of the ethics approval of the Institutional Review Board of Hong Kong Polytechnic University.
(PDF)

S3 File Proof of approval from funder. The detail of the project on the funder’s webpage (https://rfs2.healthbureau.gov.hk/app/fundedsearch/projectdetail.xhtml?id=3520).
(PDF)

S4 File Protocol. The original protocol.
(PDF)
