# Supplementary material for: Training secondary school students as anti-smoke ambassadors using the service-learning model: A cluster randomized controlled trial with hybrid type 1 effectiveness-implementation design study protocol
Source: PLoS One. 2024 Nov 14;19(11):e0313404. doi: 10.1371/journal.pone.0313404 (PMC11563479; doi:10.1371/journal.pone.0313404)
Supplement: S2 File — The letter of notification of the ethics approval of the Institutional Review Board of Hong Kong Polytechnic University. (PDF) [file pone.0313404.s002.pdf]

THE HONG KONG POLYTECHNIC UNIVERSITY
香港理工大學

To	Lam Ka Wai Katherine (School of Nursing)		
From	Pang Marco Yiu Chung, Chair, PolyU Institutional Review Board		
Email	marco.pang@polyu.edu.hk	Date	10-Jan-2023

Application for Ethical Review for Teaching/Research Involving Human Subjects

I write to inform you that approval has been given to your application for human subjects ethics review of the following project for a period from 01-Apr-2023 to 31-Mar-2026:

**Project Title:**	互學互助無煙先鋒計劃 Learning while serving: Training secondary school students as anti-smoke ambassadors using the service-learning model
**Department:**	School of Nursing
**Principal Investigator:**	Lam Ka Wai Katherine
**Project Start Date:**	01-Apr-2023
**Project type:**	Human subjects (clinical)
**Review type:**	Expedited Review
**Reference Number:**	HSEARS20221124003

You will be held responsible for the ethical approval granted for the project and the ethical conduct of the personnel involved in the project. In case the Co-PI, if any, has also obtained ethical approval for the project, the Co-PI will also assume the responsibility in respect of the ethical approval (in relation to the areas of expertise of respective Co-PI in accordance with the stipulations given by the approving authority).

You are responsible for informing the PolyU Institutional Review Board in advance of any changes in the proposal or procedures which may affect the validity of this ethical approval.

Pang Marco Yiu Chung

Chair

PolyU Institutional Review Board
