# Supplementary material for: Training secondary school students as anti-smoke ambassadors using the service-learning model: A cluster randomized controlled trial with hybrid type 1 effectiveness-implementation design study protocol
Source: PLoS One. 2024 Nov 14;19(11):e0313404. doi: 10.1371/journal.pone.0313404 (PMC11563479; doi:10.1371/journal.pone.0313404)
Supplement: S3 File — The detail of the project on the funder’s webpage (https://rfs2.healthbureau.gov.hk/app/fundedsearch/projectdetail.xhtml?id=3520). (PDF) [file pone.0313404.s003.pdf]

Research Fund Secretariat
Health Bureau
The Government of the Hong Kong Special Administrative Region
of the People's Republic of China

Detail of Approved Project (Reference No.: 06210048)

**Fund**	: HMRF - Health Promotion
**Project Status**	: Current
**Reference No.**	: 06210048
**Project Title**	: Learning while serving: Training secondary school students as anti-smoke ambassadors using the service-learning model
**Research Activity Code**	: Prevention of disease and conditions, and promotion of well-being
**Health Category**	: Generic health relevance
**Applicant(s)**	: LAM Katherine Ka-wai[1]<br>HO Ka Yan[2]<br>MAK Yim Wah[2]<br>LEUNG Doris Yin Ping[2]<br>WONG Arkers Kwan Ching[1]<br>WU Cynthia[1]
**Affiliation(s)**	: School of Nursing, The Hong Kong Polytechnic University[1]<br>School of Nursing , The Hong Kong Polytechnic University[2]
**Approved Amount (HK$)**	: $727,400.00
**Abstract**	: Objective: This project aims to promote smoking cessation by training secondary school students as anti-smoke ambassadors (ASAs) with increased knowledge, skills and self-efficacy on smoking cessation and AWARD (Ask, Warn, Advise, Refer, Do-it-again) model using service-learning model. Hypothesis: ASAs will report statistically significant higher level of knowledge, self-efficacy, and improved practice and attitude in smoking cessation and AWARD model at follow-up when compared to baseline. Design and subjects: This is a health promotion project to recruit secondary school students (Forms 2 to F4) in Hong Kong as ASAs. Interventions: Recruited students will attend a 3-hour training workshop, followed by hands-on sessions supervised by trained nursing students in Youth Quitline, then a 6-month smoker referral competition and an award presentation ceremony. Instruments: Questionnaires which were previously used in our team’s projects on smoking cessation and semi-structured interviews will be used. Main outcome measures: The number of ASAs trained and smokers being delivered the brief interventions, ASAs’ level of knowledge, practice, attitude and self-efficacy in smoking cessation and AWARD model immediately after the training programs and 3, 6, and 12 months after the programs. Data analysis: Descriptive statistics, one-way repeated measures ANOVA, logistic regressions, and content analysis will be used. Expected outcomes: At least 360 secondary school students will attend the training programs and be trained as ASAs. The project will enhance the community capacity in smoking cessation by training a considerable number of ASAs who will deliver brief interventions to numerous smokers when they encounter smokers.
**Keywords**	:
**Instruments**	:
**Remarks**	:
**Dissemination Report**	:
**Final Report**	:

Add to Bookmark Back

2013© | Important Notices | Privacy Policy
