# Supplementary material for: Training secondary school students as anti-smoke ambassadors using the service-learning model: A cluster randomized controlled trial with hybrid type 1 effectiveness-implementation design study protocol
Source: PLoS One. 2024 Nov 14;19(11):e0313404. doi: 10.1371/journal.pone.0313404 (PMC11563479; doi:10.1371/journal.pone.0313404)
Supplement: S4 File — The original protocol. (PDF) [file pone.0313404.s004.pdf]

(a) Justification for Conducting the Project:

(i) Health needs of the local community (supported by published data or relevant experiences)

Smoking remains the leading cause of preventable premature death and illness worldwide,[1] accounting for more than 8 million deaths annually.[1,2] Evidence shows that smoking cessation interventions in young people are crucial for the tobacco endgame,[3,4] especially as smoking behavior from youth continues into adulthood.[9] Early smoking cessation interventions are pivotal.

Youth Quitline (YQL)[5] is the only existing telephone hotline funded by the Department of Health in Hong Kong. It provides free peer smoking cessation counseling to young smokers aged 25 years or younger. YQL is currently operated by our research team members (KKWL, KYH, YWM, YPL, and CW). YQL represents a major success when compared with earlier results and other smoking cessation services in Hong Kong.[6,7] However, YQL recruited smokers through outreach activities and referrals from non-governmental organizations (NGOs). Many smokers have already smoked for 2 years or more at the time of recruitment. Given that early interventions are important for tobacco endgame[3,4] and most daily smokers started smoking weekly at age 10–19 years,[8] alternative methods for recruitment has to be considered. Youngsters encounter peer smokers frequently and easily in their social network. Therefore, **building the community's capacity by training youngsters to refer smokers to professional smoking cessation services offers a good strategy to support smoking cessation**. Additionally, the trained youngsters will be educated about not smoking.

Peer pressure is considered the most common reason for smoking in young people.[8,9] **Smoking cessation interventions that target peers are likely to have substantial effects**. The second most common reason reported is **curiosity**.[8] This suggests that if young people clearly understand the consequences of smoking, they may be less likely to smoke because of curiosity. Evidence also indicates that youngsters may not have adequate knowledge about smoking and its consequences, which results in myths about smoking.[10] Once smoking behavior is initiated, individuals are easily addicted, which results in difficulty in quitting, thereby carrying smoking behavior into adulthood and later life.[9] Therefore, **education to resolve these myths** is particularly important.

(ii) Literature review on others' strategies to address these needs

A literature review revealed that **brief interventions** and **referrals** are important strategies to assist smokers to receive professional help to quit smoking successfully, as supported by the World Health Organization (WHO) and our previous publications.[11-13] Particularly, the **AWARD** (***A***sk, ***W***arn, ***A***dvise, ***R***efer, ***D***o-it-again) model emphasizes referrals and offers a brief and effective intervention for smoking cessation.[6,12] The Department of Health is a significant organization that delivers numerous smoking cessation health talks in the community. Another core annual activity organized by the Council on Smoking and Health and the Education Bureau in Hong Kong is the **Smoke-free Elite Teens Program** (formerly known as the Smoke-free Teens Program).[14,15] Our research team members (KKWL and KYH) were previously involved in assisting with the program delivery. This program aims to nurture young people aged 14–18 years to promote a smoke-free culture in Hong Kong by firstly teaching participants the AWARD model and updated information on tobacco control, then requiring them to organize smoke-free programs in the community.[14,15] The program has gained support from over 130 secondary schools, youth centers, and uniform groups. Nevertheless, the referrals rate was low with merely 1% of participants had referred smokers to smoking cessation services after the program.[14] One key reason is that the program did not provide hands-on experience to participants to proactively approach smokers and use the AWARD model. Most of them reported that

they felt embarrassed and did not know how to effectively advise smokers to quit or refer smokers to professional services. Evidence also shows that hands-on experience is needed to effectively deliver learned skills.[16] To fill this service gap, **our proposed health promotion project based on the service-learning model will provide opportunity for young people to learn and practice the AWARD model with hands-on experience under the supervision of nursing students who are considered experienced peer counselors.**

(iii) Scientific evidence supporting the strategies to address these needs proposed in this project

AWARD model

The AWARD model involves an effective five-step process for smoking cessation when an individual encounters smokers: (1) ***A***sk about smoking history, (2) ***W***arn about the high risk of smoking, (3) ***A***dvise to quit; (4) ***R***efer smokers to smoking cessation services; and (5) ***D***o it again.[6,12] It emphasizes referral (i.e., referring smokers to existing smoking cessation services), which has been found to assist smokers to quit successfully.[17] This model is particularly suitable for young people as it is easily learned and used with minimal training.[6,12] Additionally, it takes only a minute or slightly longer to deliver smoking cessation advice based on the AWARD model.[17] Thus a large number of smokers can be reached at low cost.[12]

Brief interventions using the AWARD model have been found to effectively assist smokers to quit and build community capacity for smoking cessation. Our team members (KKWL and KYH) trained 50 girl guides to promote smoking cessation among community-living female smokers using the AWARD model which only lasted for a minute or slightly longer.[12] The findings showed that the intervention was feasible and effective in promoting smoking cessation among community-living female smokers.[12] However, that intervention only targeted training female teenagers to refer female smokers,[12] whereas most smokers in Hong Kong are male.[8]

Service-learning model

The service-learning model provides young people with opportunities to gain hands-on experience in using the AWARD model. [18,19] This model has been used by the Hong Kong Polytechnic University (PolyU) over the past decade in service-learning subjects. Service-learning is a type of experiential education that includes two fundamental constructs: service and learning.[20] The model requires senior or experienced workers who can monitor and supervise students in conducting activities when serving the community. It has been documented to benefit students by facilitating their intellectual and academic development and building their sense of civic responsibility through reflections and applying knowledge learned in classroom to real-world practice.[20] The model has also been published by our team[19] and is currently being used to operate YQL. Under this model, **all nursing students in year 3 of bachelor's or mental health programs** are trained as peer counselors and receive supervision by nursing teachers while delivering smoking cessation counseling in their clinical placement in YQL. Year 3 students are chosen as they have gained relevant knowledge about counseling and addictive behavior in their previous years of study. Over a 7-month period, the students referred 3,142 smokers to YQL and provided smoking cessation counseling to 336 youth smokers. This represented a remarkable success, as a previous evaluation study indicated that YQL had provided counseling to 1,684 youth smokers over a 10-year period before adopting this model.[6]

(b) Aim and Objectives:

This project aims to promote smoking cessation by training young people as anti-smoke ambassadors (ASAs) with increased knowledge on smoking cessation and skills using the AWARD model to build community capacity in smoking cessation. The objectives are to: (1) examine the effectiveness of our program in assisting secondary school students to refer smokers to YQL (primary objective), (2) increase ASAs' knowledge about smoking cessation and skills using the AWARD model, (3) enhance their practice and attitudes toward smoking cessation and AWARD model, (4) increase their self-efficacy in using AWARD model skills, (5) provide hands-on experience in using the AWARD model through the service-learning model, (6) train ASAs to deliver brief interventions to young smokers using the AWARD model, and (7) identify facilitators and barriers in implementing in the real-world.

(c) Project Plan:

[Please state clearly the pre-set criteria for process and outcome evaluation based on the RE-AIM framework. Please refer to https://re-aim.org/ or https://www.fic.nih.gov/About/center-global-health-studies/neuroscience-implementation-toolkit/Pages/methodologies-frameworks.aspx for reference.]

[For Seed Grant application (i.e. grant ceiling is HK$500,000), please state clearly the pre-set criteria to enable scale-up to a larger project and/or enhance the efficacy/effectiveness of existing practice.]

(i) Target group (justify and explain the feasibility in reaching the target group size)

Secondary school students (Forms 2–4) are the target group as most daily smokers started smoking weekly at age 10–19 years.[8] We targeted Forms 2–4 as Form 5 and 6 students usually concentrate on preparing for the public examinations, and Form 1 students may be new to secondary school. PI and Co-I (KYH) have conducted a project to promote smoking cessation among community-living female smokers by training female young smoking cessation and reduction ambassadors aged 13-18.[12] In that project, 160 Girl Guides with a mean age of 14.7 years (similar ages to students in forms 2-4) were trained as ambassadors. Among them, 50 were recruited in that project to deliver brief advice with AWARD model to unknown female smokers. 106 adult female smokers (with mean age 35.8 years) received the brief intervention. At 6-month follow-up, their self-reported abstinence was 12.2%, indicating the ability of young students to deliver smoking cessation messages to unknown smokers.

Sample size calculation

To estimate the required sample size, G*Power[21] was used based on a small-to-medium effect size on the primary outcome, i.e. number of smoker referred by secondary school students at the 6-month follow-up (d=0.35), based on our team's experience in smoking cessation projects.[12-14] This study involves a two-arm cluster randomized controlled trial (RCT) to examine the primary objective, i.e. test the effectiveness of our program in assisting secondary school students to refer smokers to YQL. Details of the study design is in (ii) Implementation plan. Hence, to detect a significant difference in the number of smoker referred between the two groups with a significance level of 5% and power of 80%, a total of 260 secondary school students would be needed. Based on discussions with some schools, a conservative estimate of around 360 eligible secondary school students can be approached in each school. The response rate for previous workshops of a similar duration was around 8% conservatively. [12, 22] Hence, we expect to recruit 28 (360 × 0.08) students in each school. For cluster RCT, it is estimated that the intracluster correlation coefficients (ICC)=0.01, and the design factor =(1+(28-1)*0.01)=1.27, then the sample size needed will be 331 (260*1.27). With an estimated 10% attrition rate at 12 months, the total sample size needed will be **368 students (331/0.9) with 184 students in each**

group. Thus, a total of 14 schools with 7 schools (184/28) in each group are needed. To recruit 14 schools with 368 students, all our team members have good connections with some other secondary schools. These schools will be invited.

We expect that the schools and students will be motivated to join is based on our experience of our team in conducting projects with secondary school students including the anti-drug program from Beat Drugs Fund (with 198 children aged 13–18)[22] and the previous project with the Girl Guide Association (with 160 Girl Guides aged 13-18).[12] In these 2 projects, schools were motivated to join as they could be recognized by the University with pennant flags. Hence, our project will also award those participating schools with pennant flags. Besides, these projects showed that students and parents were very motivated in joining health promotion activities from the University as it provided students opportunities to gain precious experience to interact with student nurses, the University and gain the health promotion knowledge. In addition, University certificates will be issued to the ASAs who completed the training workshops with hands-on sessions. From the two aforementioned projects, students and parents mentioned to value the certificates and express to be very willing to join similar health promotion activities again in the future.

(ii) Implementation plan

The project will be completed in 3 years.

Design

A hybrid type 1 effectiveness-implementation design will be adopted as it can primarily focus on testing the effectiveness of our intervention on the relevant outcomes while observing and gathering information on implementation.[23] This can allow us to identify barriers, facilitators, and what is required for supporting the implementation in the real world and therefore inform the appropriate implementation strategies.[23] With the hybrid type 1 design, our main objective is to examine the effectiveness of the intervention. Information relating to implementation will also be studied as secondary outcomes. To do so, a two-arm cluster RCT following the CONSORT statement in Appendix 1) will now be conducted to examine the effectiveness of the our program in referring smokers to YQL. The Reach Effectiveness Adoption Implementation Maintenance (RE-AIM) framework will also be adopted.

Clusters and randomization

14 clusters (14 schools) will be randomized to the intervention or control group using a 1:1 ratio by a RA who is not from our team using an independent computerized randomization sequence process that can only be accessed by that RA to ensure allocation concealment. This RA will login to an online platform to perform randomization. Randomization sequences will be generated by the computer in randomly permuted blocks sizes of 2, 4, and 6. The RA responsible for recruitment will record the exact date and time of recruitment. Computerized time stamps and electronic logs of sent allocation will be used to ensure allocation concealment. The RA undertaking recruitment will be responsible for data collection and be blinded to the group assignment.

Intervention group (ASAs)

Secondary school students will be required to join our 3-phase ‘Learning while serving’ program.

<u>Phase 1: ASA recruitment</u>

This involves establishing a network for smoking cessation in the community. It includes recruiting ASAs through secondary schools. Promotional leaflets will be designed and distributed to the two NGOs and other secondary schools. Eventually, a community network with young people ready to be trained as ASAs for smoking cessation will be established.

Phase 2: Training program

The training program will involve **training workshops**, **hands-on sessions,** and **a smoker referral competition**.

1. Training workshops

Our team will develop a half-day workshop (Appendix 2) to train ASAs, aiming to equip the established community network with relevant knowledge and skills in delivering brief interventions using the AWARD model to promote smoking cessation among smokers in Hong Kong. Each workshop will be conducted by a research assistant (RA) trained by our team. The workshop will last for 3 hours and be held in secondary schools, at PolyU, or online (depending on the pandemic situation and preference of schools). Workshops will be held on Saturdays or Sundays to avoid clashing with school classes. During the summer, workshops will also be held on weekdays. The workshops will be based on those implemented in our previous smoking cessation projects.[6,12,14] Specifically, when teaching the AWARD model, our team will focus on educating ASAs about the skills needed to assess smokers' smoking status, readiness to quit, and nicotine dependence. Importantly, ASAs will be taught how to deliver a brief intervention using the AWARD model (lasting about a minute or slightly longer) (Appendix 3).[12] Information of YQL will be highlighted so that ASAs can invite smokers to call YQL in '***R***efer' of the AWARD model.[12] Also in '***R***efer' of the AWARD model, the ASAs will be trained with some safety tips, which are the same as that taught in nursing students of YQL, by the RA. Particularly, the RA will educate ASAs about safety tips in conducting outreaching activities in the hands-on sessions, including (1) stay with the respective nursing students at all times during the outreaching sessions, (2) stay on main streets only and never go to alleys, (3) never reveal your identity cards/student cards/telephone numbers or other documents/numbers that reveal your identities, (4) report to nursing students if you feel unwell/ any discomfort, (5) introducing signals of discord, and (6) educating appropriate solutions dealing with signals of discord. Session 5 and 6 about signals of discord will also be particularly useful for ASAs who may encounter and offer advice to those who are older than ASAs or less amenable to receiving an intervention or get annoyed. Signals of discord is based from Motivational Interviewing (MI) which is an effective and evidence-based counselling technique for smoking cessation.[24] Signals of discord are the signals or statements that represent disharmony, such as anger, defending, squaring off, interrupting, and disengagement.[24] According to MI, examples of solutions dealing with these signals are apologizing, affirming, shifting focus, and reframing.[24] These solutions will be taught by lectures with demonstrations and ASAs will be required to role-play with each other and discuss in '**R**efer' of the AWARD model of the training workshops.

Each ASA will be required to pass an examination before proceeding to the hands-on sessions, which will confirm that they are capable of delivering the brief intervention to smokers using the AWARD model. This examination has been adopted in our previous projects.[6,12] It will first involve a written test asking ASAs about their knowledge of smoking consequences and AWARD model, then an oral demonstration where role-play assessments will be conducted. During the oral demonstration, each ASA will be required to role-play to deliver the brief smoking cessation intervention using the AWARD model in response to a case scenario of a smoker (role-played by nursing students trained in YQL). The examinations will be assessed by our RA and team members, with 50% as the passing mark. Those who fail will be required to rejoin the training workshops and retake the examination until a passing mark is obtained.

2. Hands-on sessions

ASAs will then be required to follow their assigned nursing students **(in year 3 of the bachelor's or mental health program)** to implement the brief interventions to smokers

in Hong Kong. These sessions are particularly incorporated to further increase the self-efficacy of ASAs in conducting the brief interventions. This will involve the use of the service-learning model and be conducted in YQL, which is already implementing the same service-learning model with the nursing students. The nursing students will be trained and receive supervision from nursing teachers while delivering smoking cessation counseling through YQL in their clinical placement.[5,6,20] This model will also be applied into the ASAs, meaning **ASAs can deliver the brief intervention using the AWARD model under the supervision of nursing students who are considered experienced counselors**. Each nursing student is expected to supervise about 1 to 2 ASAs based on random assignment by YQL staff during their nursing clinical placement, which lasts for about 2 months. During their placements, they are required to conduct outreach activities, which are implemented in various smoking hotspots (i.e., places where smokers gather to smoke, such as Causeway Bay and Mongkok trash cans) all over Hong Kong, and counseling for smokers, which are at the YQL center in PolyU. Specifically, nursing students will bring their ASAs to the outreach activities (2 hours each) to **supervise ASAs delivering brief interventions using the AWARD model when encountering smokers aged 25 or below smoking.** Each ASA will follow their respective nursing student to four outreach activities and two counseling sessions (2 hours each). During the counseling sessions, ASAs will listen to how the nursing students deliver smoking cessation counseling and join the conversations by applying the AWARD model, offering ASAs opportunity to experience smoking cessation counseling. All ASAs will receive a certificate after completion of this phase.

3. Smoker referral competition

All ASAs will then be given a 6-month period to use the AWARD model to deliver brief interventions to refer smokers who are aged 25 years or below (corresponding to the YQL age limit) to YQL independently, without the help of nursing students. To ensure ASAs' safety, **they are asked to approach smokers via their social network including peer smokers, smokers in families and schools**. Our team will remind ASAs before the competition that due to safety, we invite them to approach smokers aged 25 or below in their social circles only.

Smokers are defined as those who smoked tobacco products (including electronic cigarettes) in the previous 30 days, as defined in YQL and other projects related to smoking.[6,12] Particularly, e-cigarette use is becoming more common in Hong Kong recently (prevalence was 0.1% in 2019 and 0.3% in 2021),[8,25] particularly in youngsters in secondary schools (secondary school students' prevalence was 0.8% in 2019 and 1.1% in 2021).[8,25] Hence, this project will also include e-cigarette smokers.

Near the end of the brief intervention with the AWARD model, ASAs will provide the contact of YQL and invite the smokers to call YQL. Those who successfully contacted YQL staff will be regarded as a successful referral case.

Monitoring brief intervention adherence

To ensure consistency of the brief interventions, all ASAs will be asked to adhere to the intervention protocol and complete the checklist developed by our team (Appendix 3) after each delivery of the brief intervention. Additionally, each ASA will be supervised by a trained nursing student. ASAs will be encouraged to share any difficulties in delivering the brief interventions to the responsible nursing student for discussion and feedback. During the competition, ASAs will also be recommended to consult our RA if they encounter any problems.

Safety issues

PI and Co-I (KYH) have conducted a similar project to promote smoking cessation among but on community-living female smokers by training female young smoking cessation and

reduction ambassadors aged 13-18.[12] During that project, we did not receive issues relating to the difficulties in delivering messages to older smokers or messages not being well-received, smokers less amenable to receiving an intervention or get annoyed.

To further ensure the safety of students, both ASA and our nursing students will be trained related skills to assist them deliver advice to adults who are much more senior them themselves. Particularly, when teaching '***R***: Refer' in AWARD model, the research assistant will educate ASAs about safety tips in conducting outreaching activities, including (1) stay with the respective nursing students at all times during the outreaching sessions, (2) stay on main streets only and never go to alleys, (3) never reveal your identity cards/student cards/telephone numbers or other documents/numbers that reveal your identities, (4) report to RA if you feel unwell/ any discomfort, (5) introducing signals of discord, and (6) educating appropriate solutions dealing with signals of discord. These tips are also taught to nursing students by the YQL staff before their placement in YQL. In addition, in the outreaching activities, we will only ask them to pair up to actively approach peers aged 25 or below to deliver brief interventions and refer the smokers to Youth Quitline (YQL). A YQL staff will also be present in each outreaching activity to monitor the students and provide assistance when necessary. Besides, in the smoker referral competition, ASAs will be asked to actively deliver the messages to smokers aged 25 or below in their social circles only, but not to unknown smokers, which can further reduce the possibility of any potential conflicts. Both nursing students and ASAs will be given a contact of the PI for emergency contact. Although the chance of occurring adverse events is expected to be minimal, a protocol for adverse events management is developed by our team to make referrals to social workers or psychiatrists or psychologists upon participants' consent (Appendix 4).

ASAs are given opportunities to practice AWARD model with our nursing students in YQL through approaching unknown smokers on streets in the outreaching activities as we have found from YQL that outreaching activities can enable approaching a huge number of smokers in a short period of time, providing ASAs with numerous practice opportunities. According to the unpublished data from YQL recently, from March 2022 to August 2022, YQL has approached over 800 smokers in their outreaching activities. This huge number is very unlikely to be reached through families, peers, and schools. Hence, bringing ASAs to outreaching activities to practice AWARD model with our nursing students in YQL is included into this project.

Phase 3: Award presentation ceremony

10 award certificates will be given to the top 10 ASAs who deliver brief interventions using the AWARD model to the largest number of smokers (excluding smokers found with the nursing students). The ASA who refers the largest number of smokers will be the champion and receive a HKD4000 book coupon. The first- and second- runner up of the ASAs will respectively receive HKD 3000 and HKD2000 book coupons. The remaining 7 ASAs will each receive a HKD1000 book coupon. Media will be invited to attend the ceremony to spread the success of ASAs and anti-smoking messages to the community.

Control group

Secondary school students will be only required to attend a 3-hour training workshops with AWARD model in their schools or the University by our trained RA. The contents of the workshops are the same as that in the intervention group. They will be introduced YQL and invited to refer young smokers aged 25 or below to YQL. However control group will not join the examinations, hands-on sessions, the smoker referral competition nor be given checklists for conducting the brief interventions with AWARD model. The activities in the control group are regarded as usual care since the training workshops are also conducted regularly by YQL staff to other secondary or tertiary schools.

Data collection and ethical considerations

Ethical approval is obtaining from the PolyU Institutional Review Board. After randomization, responsible teachers in schools will first be briefed by our RA about the purpose and protocol of the project. The schools will then distribute our promotional leaflets, information sheet and consent forms to students or parents depending on schools' preferences. Consent forms with students'/parents' telephone numbers will be returned to teachers if students are interested in joining the project. Eligible students will be informed by our RA via telephone that their participation will be voluntary and confidentiality will be maintained. They will also be notified about the details and schedule of the program activities. The RA will contact the students in the intervention and control group at the start of training program (T1), at the end of training program (T2), and at 3 (T3), 6 (T4), and 12 months (T5) after the end of the training program by telephone to complete a set of questionnaires. These time points were chosen as they are commonly used in health promotion projects to monitor the effects of outcomes.[12,19]

(iii) Contingency / alternative plan if any problem encountered during implementation

Program activities may be conducted online if the pandemic situation become serious so that face-to-face mode can be compared with online mode to see which might work best.. Additionally, if fewer than 90 secondary school students or 7 schools are recruited within 4 months, our team will approach more secondary schools and NGOs for recruiting young people.

(iv) Cross-sector collaboration

We will collaborate with secondary schools in Hong Kong to recruit secondary school students. We will also collaborate with YQL to ensure ASAs have hands-on experience of using the AWARD model skills in smoking cessation with the supervision of trained nursing students.

(v) Indicators and targets

This project involves repeated measures and mixed-method study design with both quantitative (evaluating reach, effectiveness, adoption, and maintenance) and qualitative (evaluating effectiveness, implementation and maintenance) approaches.

Indicators

The Reach Effectiveness Adoption Implementation Maintenance (RE-AIM) framework will be adopted. All indicators are proposed according to our previous experience on smoking cessation.[12-14,19]

1. Reach (quantitative) will be recorded as: (1) the number and proportion of secondary schools, (2) secondary school students, and (3) nursing students reached; (4) the number of workshops conducted; (5) the numbers of ASAs in the workshops and that passed the examinations (involving a written test and an oral demonstration; the oral examination will be assessed through role-play assessments); (6) the number of outreach sessions conducted; (7) the number of smokers reached in the outreach sessions; (8) the number of counseling sessions in which ASAs were involved; and (9) the number of smokers that the ASAs involved in counseling sessions. Indicators from (7) to (9) will be recorded in log sheets with the exact time and date by ASAs. Other numbers will all be recorded in log sheets with the exact time and date by the RA as in YQL. All indicators here will be verified with YQL staff.
2. Effectiveness (quantitative and qualitative) will be recorded in terms of: (1) the number of smokers refer by secondary school students to YQL (Smokers who have contacted YQL and this will be verified with YQL staff) (primary effectiveness measure), (2) the level of secondary school students' knowledge in smoking cessation and AWARD model, (3) practice (such as asking the secondary school students the number of times delivering the brief intervention and the number of smokers who receive their brief

interventions in question D12) and attitudes toward smoking cessation and AWARD model, (4) self-efficacy in using AWARD model. The scales have all been used in the project with Girl Guide Association[12], Smoke-free Elite Teens Program,[14,15] and YQL.[19] The primary outcome will be the number of smokers who referred by secondary school students to YQL at T4 (at the end of the 6-month competition). The secondary school students will be asked to deliver the brief interventions with AWARD model and then ask the smokers to call YQL hotline towards the end of the intervention. Those who successfully contacted YQL will be counted as a successful referral.

3. Adoption (quantitative): the numbers and proportions of (1) ASAs and (2) nursing students adopting the brief interventions with AWARD model to smokers. The number of ASAs who join the smoker referral competition will be recorded by the RA in log sheets with the exact time and date. The intention of ASAs and nursing students to conduct the brief interventions with AWARD model in the future at T2-T5 will also be assessed in questionnaires.
4. Implementation (qualitative): (1) facilitating factors, (2) barriers, and (3) feasibility for implementing the project. These factors will be explored using semi-structured interviews with the responsible teachers in the participating secondary schools, ASAs, participating nursing students, and responsible staff in YQL at T2.
5. Maintenance, or the effectiveness of the project in long term will be measured at T5 (quantitative and qualitative): (1) the number of smokers referred to YQL by secondary school students (verified with YQL staff), (2) the level of secondary school students' knowledge in smoking cessation and the AWARD model, (3) practice and attitude towards smoking cessation and AWARD model, and (4) self-efficacy in using AWARD model. At the setting level, the components that were maintained in the project at T5 will be assessed using semi-structured interviews with ASAs and participating nursing students.

Targets/pre-set criteria for evaluations

The targets are set based on our team's experience from YQL[19] and in conducting smoking cessation and health promotion projects.[12-14, 22]

1. Reach: (1) 14 (100%) secondary schools, (2) at least 5,040 secondary students (30 students each class x4 classes x3 Forms x14 schools), and (3) at least 184 nursing students. (4) At least 7 training programs will be conducted (around one training program in each school x7 schools in the intervention group), with (5) at least 184 ASAs attending the training programs. (6) Each ASA will attend at least two outreach sessions, and (7) at least 368 smokers will be reached in the outreach sessions. (8) Each ASA will be involved in at least one counseling sessions, with (9) at least 184 smokers reached in the counseling sessions.
2. Effectiveness: The primary hypothesis is that ASAs will report a statistically significant (1) larger number of smokers refer to YQL at T2 than that in the control group. Secondary hypotheses are: ASAs will report a statistically significant (2) higher level of secondary school students' knowledge in smoking cessation and AWARD model, (3) improved practice (such as asking the secondary school students the number of times delivering the brief intervention with AWARD model and the number of smokers who receive their brief interventions in question D12) and attitudes toward smoking cessation and AWARD model, (4) higher level of self-efficacy in using AWARD model, than that in the control group at T2–T5. If the project can achieve the primary hypothesis, it will be considered as effective.
3. Adoption: at least (1) half of the ASAs and (2) 70% of the nursing students will intend to use the brief intervention for smokers in the future at T4.

4. Implementation: (1) facilitating factors and (2) barriers will be identified at T2. (3) It will be feasible to implement the project in secondary schools and YQL.
5. Maintenance: we hypothesize that ASA will report statistically significantly higher (1) number of smokers referred to YQL at T5 when compared to T2 and the control group. We also hypothesize that ASAs' (2) level of knowledge about smoking cessation and the AWARD model, (3) practice and attitude towards smoking cessation and the AWARD model, and (4) self-efficacy in using the AWARD model at T5 will be maintained when compared with T2, and statistically significantly higher than that in the control group. At the setting level, at least 70% of the components will be maintained in the project at T5.

(vi) Evaluation plan

Questionnaires will be used in Effectiveness of REAIM. These questionnaires were **commonly and previously adopted and validated in smoking cessation projects** including the project with Girl Guide Association[12], Smoke-free Elite Teens Program,[14,15] and YQL.[19] Semi-structured interviews based on an interview guide developed by our team and ASA's reflective journals will be used. Only those smokers who successfully contacted YQL will be counted as a successful referral case.

Outcomes assessed in questionnaires developed by our team

1. **Demographic data** including age, sex, household size, household income, living district, and smoking status of the secondary school students and participating nursing students will be collected at T1.
2. **Secondary school students' level of knowledge in smoking cessation and the AWARD model** will be assessed by asking ASAs to respond to 19 related items at T1–T5.
3. **Secondary school students' practice and attitude towards smoking cessation and the AWARD model** will be recorded by asking ASAs to respond to 31 related items at T1–T5.
4. **Secondary school students' self-efficacy in using the AWARD model** will be assessed by asking ASAs to answer three items (scale 1–10) at T1–T5.

Questionnaires number 2 to 4 are used in Effectiveness of REAIM and **commonly and previously used and validated in smoking cessation projects** including the project with Girl Guide Association[12], Smoke-free Elite Teens Program,[14,15] and YQL.[19]

Semi-structured interviews and reflective journals

Each ASA will write a 300-word reflective journal at T2. Responsible teachers in participating secondary schools, participating nursing students, ASAs, and responsible YQL staff will be randomly invited to complete a one-to-one semi-structured interview focused on implementation. The interviews will be conducted by an RA at T2 until data saturation is reached. ASAs and nursing students will be interviewed about maintenance at T5. Another RA who is not involved in this project will be asked to generate random numbers using a computer for random selection of participants for the interviews. All interviews will last for around 15–30 minutes and be audio-recorded in a room in our university or online (e.g., via Zoom) depending on the preference of interviewees. Based on the interview guide, they will be asked about their overall experience, facilitating factors, barriers, and feasibility of implementing brief interventions using the AWARD model to smokers at T2. At T5, they will be asked about the maintained effects and components of the project. It is estimated that around 40 and 20 interviewees will be needed to reach data saturation at T2 and T5, respectively. Each interviewee will receive HKD$200 as a travelling allowance.

(vii) Results analysis

Quantitative data will be analyzed using SPSS version 28. Descriptive statistics will be reported (objectives 5 and 6). The characteristics of ASAs and nursing students who intend to use the AWARD model in the future will be compared with those who do not (adoption) using independent sample t-tests and chi-square tests (objective 6). Mixed between-within-subjects analysis of variance and odds ratios with logistic regression will be used to examine the differences in outcomes between the ASAs and the control group (objectives 1–4) at each follow-up. Intention-to-treat analyses and multiple imputation will also be used.

QSR NVivo version 12 will be used to organize the qualitative data from the interviews (objective 7) and reflective journals. ASA's reflective journals will be particularly analyzed for the effects of our program in helping ASAs to refer smokers to YQL, ASAs' level of knowledge, practice and attitude in smoking cessation and the AWARD model, and self-efficacy in using the AWARD model (objective 1-4). Content analysis will be performed by two team members (KWKL and KYH) separately by applying bracketing. Descriptions will be returned to participants for checking and feedback. Any discrepancies will be discussed and resolved in regular meetings among team members.

(d) Key References:

1. World Heath Organization. Tobacco facts. Accessed January 21, 2022. https://www.who.int/news-room/fact-sheets/detail/tobacco
2. Institute of Health Metrics. Global Burden of Disease. Accessed January 19, 2022. http://ghdx.healthdata.org/gbd-2019.
3. Coombs J, Bond L, Van V, Daube M. "Below the Line": The tobacco industry and youth smoking. *Australas Med J.* 2011;4(12):655-673.
4. Gendall P, Hoek J, Marsh L, Edwards R, Healey B. Youth tobacco access: trends and policy implications. BMJ Open. 2014;4(4):e004631.
5. Hong Kong Polytechnic University. Youth Quitline. Accessed January 19, 2022. https://www.polyu.edu.hk/en/media/media-releases/2021/0302_polyu_sn_youth_quitline/
6. Li WHC, Chan SSC, Wang MP, Ho KY, Cheung YTY, Chan VWF, et al. An Evaluation of the Youth Quitline Service Young Hong Kong Smokers. *J Adolesc Health.* 2017;60(5):584-591.
7. Chan SS, Wong DC, Cheung YT, et al. A block randomized controlled trial of a brief smoking cessation counselling and advice through short message service on participants who joined the Quit to Win Contest in Hong Kong. *Health Educ Res.* 2015;30(4):609-621.
8. Census and Statistics Department. Thematic Household Survey Report No. 70: Pattern of smoking. Accessed January 19, 2022. https://www.censtatd.gov.hk/en/data/stat_report/product/B1130201/att/B11302702020XXXXB0100.pdf
9. Chassin L, Presson CC, Sherman SJ, Edwards DA. The natural history of cigarette smoking: predicting young-adult smoking outcomes from adolescent smoking patterns. *Health Psychol*. 1990;9(6):701-716.
10. Loke AY, Wong YP. Smoking among young children in Hong Kong: influence of parental smoking. *J AdvNurs*. 2010;66(12):2659-2670.
11. World Health Organization (WHO). Brief intervention for substance use: Brief intervention for substance use: A manual for use in primary care. Accessed February 21, 2022. https://www.who.int/substance_abuse/activities/en/Draft_Brief_Intervention_for_Substance_Use.pdf
12. Ho KY, Li WHC, Lam KKW, et al. Promoting smoking cessation among community-living female smokers by training smoking cessation and reduction ambassadors. *J Public Health (Oxf).* 2020;42(1):53-61
13. Li WHC, Ho KY, Wang MP, et al. Effectiveness of a Brief Self-determination Theory-Based Smoking Cessation Intervention for Smokers at Emergency Departments in Hong Kong: A Randomized Clinical Trial. *JAMA Intern Med.* 2020;180(2):206-214.
14. Chung OK, Li WHC, Ho KY, et al. A descriptive study of a Smoke-free Teens Programme to promote smoke-free culture in schools and the community in Hong Kong. *BMC Public Health*. 2019;19(1):23.

15. Council on Smoking and Health. Smoke-free Elite Teens Programme. Accessed January 21, 2022. https://www.smokefree.hk/page.php?id=47&lang=en
16. Agaku IT, Olaiya O, Quinn C, et al. A Mixed-Methods Assessment of a Brief Smoking Cessation Intervention Implemented in Ohio Public Health Clinics, 2013. *Matern Child Health J.* 2015;19(12):2654-2662.
17. Clinical Practice Guideline Treating Tobacco Use and Dependence 2008 Update Panel, Liaisons, and Staff. A clinical practice guideline for treating tobacco use and dependence: 2008 update. A U.S. Public Health Service report. *Am J Prev Med*. 2008;35(2):158-176.
18. Kerrissa H. Fundamentals of Service-Learning Course Construction. Rhode Island: Campus Compact; 2001.
19. Ho KY, Lam KKW, Wu CST, Leung DYP, Yeung WF, Hung TM, Ting S, Tong MN, Tang LN, Mak YW. Utilization of the Youth Quitline as an opportunity for undergraduate nursing students to deliver smoking cessation counselling as their clinical placement: An implementation of a service-learning model. *Nurse Education Today.* 2022.
20. Shek DTL, Ma CMS, Yang Z. Transformation and Development of University Students through Service-Learning: a Corporate-Community-University Partnership Initiative in Hong Kong (Project WeCan). *Applied Research in Quality of Life.* 2020;15: 1375–1393.
21. Faul F, Erdfelder E, Lang AG, Buchner A. G*Power 3: a flexible statistical power analysis program for the social, behavioral, and biomedical sciences. *Behav Res Methods*. 2007;39(2):175-191
22. Ho KY, Lam KWK, Tong ETF, et al. Development of a Community-Based Network to Promote Anti-Drug Messaging and Identify Hidden Drug Abusers in Hong Kong. *Int J Environ Res Public Health*. 2022;19(18):11544.
23. Landes SJ, McBain SA, Curran GM. An introduction to effectiveness-implementation hybrid designs. *Psychiatry Res*. 2019;280:112513.
24. Miller WR, Rollnick S. *Motivational Interviewing: Helping People Change.* 3rd ed. New York Guilford Press; 2013.
25. Census and Statistics Department. Thematic Household Survey Report No. 75: Pattern of smoking. Accessed 5 November, 2022. https://www.censtatd.gov.hk/en/data/stat_report/product/C0000047/att/B11302752022XXXXB0100.pdf
